# Identifying the contributions of progenitor *Malus* species to cultivated apple (*M. domestica*) using 20K SNP array data

**DOI:** 10.1186/s12864-026-13023-z

**Published:** 2026-06-11

**Authors:** Nicholas P. Howard, Stijn Vanderzande, Gulzar Khan, James J. Luby, Gayle M. Volk, Dirk C. Albach, Cameron Peace

**Affiliations:** 1 Institut Für Biologie Und Umweltwissenschaften, Carl Von Ossietzky University, Oldenburg, 26129 Germany; 2 Department of Horticultural Science, University of Minnesota, St. Paul, MN 55108 USA; 3 Department of Plant Breeding, Wageningen University and Research, Wageningen, 6708 PB The Netherlands; 4 Affiliate Faculty, Department of Horticulture and Landscape Architecture, Colorado State University, Fort Collins, CO 80523 USA; 5 Department of Horticulture, Washington State University, Pullman, WA 99164 USA

**Keywords:** Ancestry-informative markers, Domestication, Haploblock, Haplotype, *M. orientalis*, *M. sieversii*, *M. sylvestris*, Admixture, SNP array

## Abstract

**Background:**

The domesticated apple (*Malus domestica*) is widely considered an admixture primarily involving *M. sieversii*, *M. sylvestris*, and *M. orientalis*, with possible contributions from tiny-fruited species such as *M. baccata*. Although this origin theory is well supported by genomic studies, the relative contributions of each progenitor species to cultivar genomes remain unclear, and taxonomic classifications of some *Malus* species are conflicting. The availability of new genomic tools and resources enables probing of these topics.

**Results:**

A panel of wild *Malus* accessions was genotyped using the Illumina Infinium 20K apple SNP array to generate a library of species-specific SNP and haploblock alleles. After excluding heavily admixed individuals and masking admixture haplotypes in individuals with small amounts of admixture, this library was used to clarify the ancestry of 436 wild accessions and estimate the genomic proportions attributable to *M. sieversii*, *M. sylvestris*, *M. orientalis*, and collectively, tiny-fruited *Malus* species in *M. domestica* cultivars. The results were broadly consistent with previous studies but revealed new ancestry details and enabled precise haplotype-level attributions. Previously unreported admixture and taxonomic irregularities were detected for some wild accessions. All *M. domestica* cultivars were found to be mixtures of the three primary progenitors, with some also showing contributions from tiny-fruited *Malus* species. The relative contributions varied among cultivars, with evidence that older cultivars and traditional cultivars originating in eastern Europe or Western Asia had less *M. sylvestris* ancestry than modern cultivars and traditional cultivars originating in Western Europe.

**Conclusions:**

This study provides new insights into apple ancestry and highlights the need for clarification in *Malus* taxonomy. The findings have implications for germplasm management, historical research, and genetic improvement of apple cultivars. The species-specific allele library developed here offers a valuable resource for routine admixture estimation of *Malus* accessions genotyped on the same set of SNPs.

**Supplementary Information:**

The online version contains supplementary material available at 10.1186/s12864-026-13023-z.

## Background

The prevailing theory of apple (*Malus domestica*) domestication proposes that it originated from *M. sieversii* in or near the Tien Shan Mountains in modern day Kazakhstan [1–3]. These early apples were disseminated westward along the Silk Road trade routes in antiquity, where they hybridized with *M. orientalis*, which is native to the Caucasus Mountains area, Iran, and Turkey. The resulting hybrids further admixed with *M. sylvestris *in Europe [2, 4–7]. Additional introgression from some tiny-fruited species from Asia, such as* M. baccata*, has also been reported [8]. Cultivated, “domesticated” apples subsequently spread across Europe and Russia and were introduced to the Americas, Australia, New Zealand, South Africa, Japan, China, and elsewhere through a combination of trade, exploration, and colonization [9]. Many of the most important modern commercial cultivars [10] have been developed in these newer apple-growing regions [11].

Although this origin theory is widely accepted, there is a general lack of clarity and disagreement in some cases regarding the relative genetic contributions of each progenitor *Malus* species to *M. domestica*. For example, some studies have concluded that *M. sylvestris* does not significantly contribute to the genomes of *M. domestica *cultivars (e.g., [12, 13]). There is additional uncertainty regarding whether these contributions differ substantially among cultivars genome-wide and in specific genomic regions. For example, Cornille et al*.* [2] reported less* M. sylvestris* ancestry in cider cultivars relative to dessert cultivars whereas Migicovsky et al*.* [14] reported the opposite. Recent work has recognized the contribution of* M. orientalis*to traditional Iranian cultivars [4]; however, how much influence* M. orientalis* has had on cultivars from other regions or on modern commercial cultivars is not known. Sun et al*.* [3] quantified the genetic contribution of* M. sieversii* and *M. sylvestris* to a whole-genome sequence assembly of ‘Gala’, and then extended the analysis to a group of *M. domestica* cultivars that were deep-sequenced. While this was a landmark study on the topic of investigating the progenitor species origins of apple cultivars, the study did not consider other progenitors of *M. domestica* or the possibility that the wild accessions used for attributing the progenitor species origins of the genomic regions of *M. domestica* cultivars were not actually pure species accessions. Volk et al. [15] found that many* M. sieversii* accessions considered pure specimens, including reference accessions used in DNA-based taxonomic studies, are actually admixed.

In addition to the previously described lack of clarity regarding the relative genetic contributions of the progenitor *Malus* species to *M. domestica*, the taxonomic classification and delineation of *Malus *species are often unclear or conflicting [16]. Consequently, accessions held in germplasm collections might have no, unclear, or incorrect species attributions (e.g., [15]). In addition, apple taxonomy includes numerous named species and interspecific hybrids of unclear origin [16, 17]. For example,* M. prunifolia *is regarded by some as a distinct species (e.g., [18]) but by others as a hybrid [17]. This lack of unambiguous classification of wild* Malus* germplasm obscures relationships with *M. domestica* cultivars. Furthermore, certain tiny-fruited *Malus* species are known to be ancestors of specific *M. domestica *cultivar lineages because of deliberate introgression during breeding (e.g., [19]). However, because various tiny-fruited* Malus* species have long been cultivated as ornamentals, pollenizers, rootstocks, and for dessert and culinary use, not all introgression events in recent centuries are likely to have been recorded in pedigrees. As a result, the genomes of some cultivars might contain cryptic introgression from one or more tiny-fruited *Malus* species. Overall, achieving a more complete understanding of *Malus* species attribution and of *Malus* germplasm more broadly would aid ongoing research into the genetics and history of apple domestication and improve classification of apple germplasm. The availability of new genomic tools and resources makes it possible to clarify the ancestral species composition of specific genomic regions and genome-wide for any *M. domestica* cultivar.

Methodologies to infer ancestry through genotypic analysis were pioneered in human genetics (e.g., [20, 21]) and might provide useful frameworks for similar analyses in* Malus*. One such approach involves the identification of ancestry-informative markers (AIMs) [21], which are DNA markers with alleles that are diagnostic of ancestry, being specific to one or more particular subpopulations. These AIMs are identified by comparing allele frequencies among representative groups in large-scale population genetics surveys. AIMs can also be defined as haploblocks – groups of closely linked markers [22]. By tallying the total number of ancestry-informative alleles or the lengths of ancestry-informative haplotypes, researchers have estimated both the extent of admixture and the proportion of an individual’s genome attributable to particular subpopulations (e.g., [21]). These approaches should be readily applicable to* Malus* if genome-wide genotypic data are available for species-representative accessions.

Genomic tools and resources that enable ancestry inference in *Malus *have advanced substantially in recent years [23]. A major breakthrough has been the genotyping of thousands of apple cultivars and wild germplasm accessions via the Illumina Infinium® 20 K SNP array [24] and the Affymetrix Axiom® 480 K SNP array [25] across multiple research projects (e.g., [15, 26–28]). These datasets have been extensively curated [29] within an ongoing apple pedigree reconstruction project [26], which integrates whole-genome sequences [30, 31], an integrated genetic map [32], and new genetic analysis methods [33–35]. Additionally, numerous wild* Malus* accessions have been genotyped on the Illumina Infinium® 20 K SNP array and evaluated for evidence of *M. domestica *admixture [15, 36]. Although SNP arrays are subject to ascertainment bias [37], the panels used for the 20 K and 480 K SNP arrays included diverse *M. domestica* cultivars, an accession of *M.* × *micromalus*, and a close descendant of *M.* × *floribunda* 821. Consequently, these SNP array datasets likely contain alleles and haplotypes suitable for investigating ancestral species contributions in *M. domestica* cultivars and other *Malus* species accessions. Together, the availability of these curated and integrated datasets, comparable wild *Malus* SNP data, and advanced analytical tools provide an unprecedented opportunity to investigate ancestry inference within *Malus*.

The objectives of this study were to (1) identify AIMs with *Malus* species-specific SNP and haploblock alleles and compile a library of these alleles, (2) use this library to resolve the ancestry of wild *Malus* accessions, (3) determine the species composition of some accessions with unclear or unknown species attribution, and (4) demonstrate the application of the species-specific allele library to estimate the genomic composition of *M. domestica* cultivars in terms of their primary progenitor species *M. sieversii*, *M. sylvestris*, and *M. orientalis* and more distant tiny-fruited *Malus* relatives (e.g., *M. baccata*). The overall goal of this study was to develop and validate a set of species-specific alleles suitable for estimating admixture and ancestry in cultivated and wild *Malus* accessions.

## Materials and methods

**Fig. 1:**
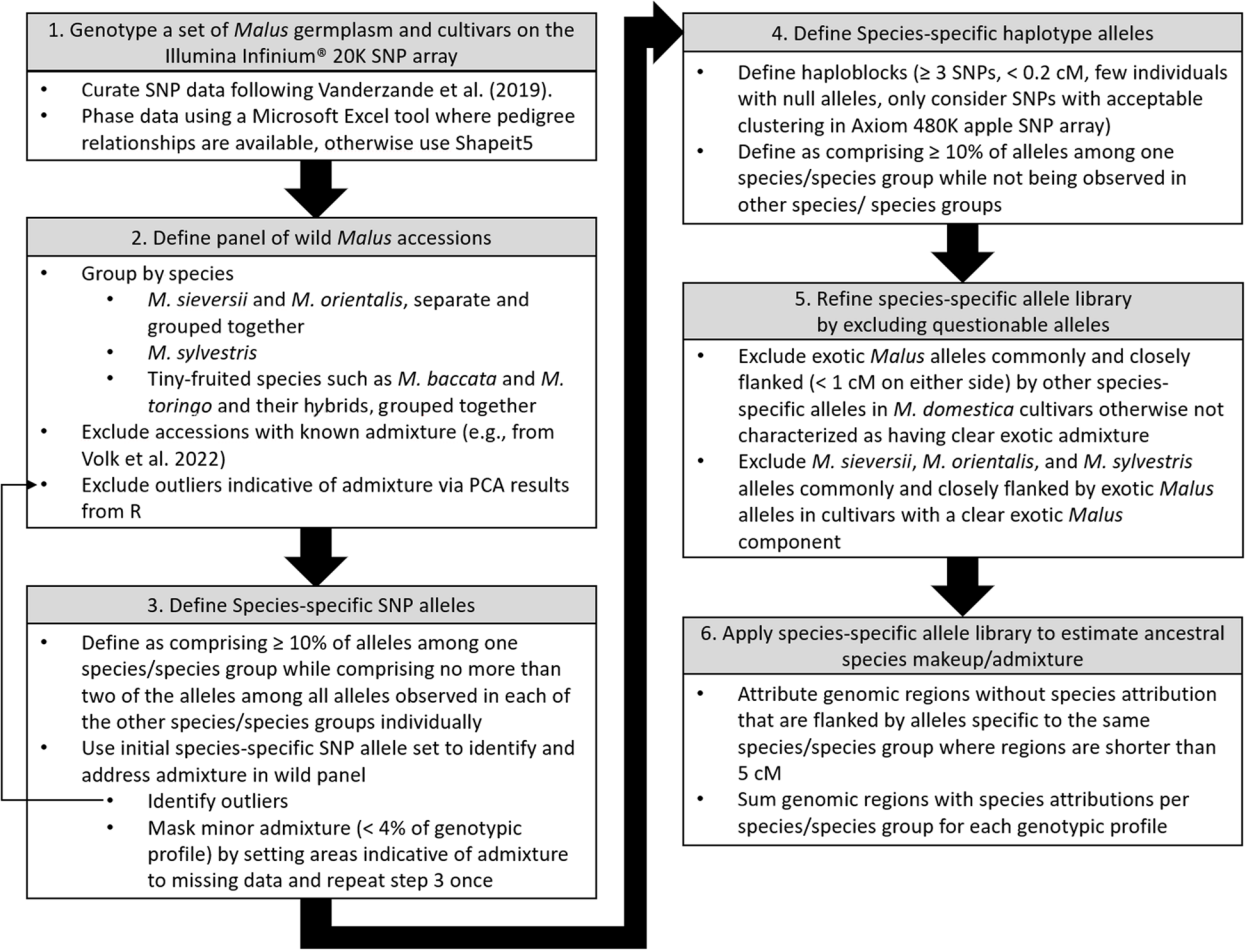
Summarized steps in the development and application of a library of *Malus* SNP and haploblock alleles

The general workflow for the creation of a species-specific allele library and its application to cultivars and accessions is depicted in Fig. 1 and is further described below.

### Germplasm and genotypic data

Accessions classified as wild *M. sieversii* (*n* = 153), *M. sylvestris* (*n* = 208), and *M. orientalis* (*n* = 32) initially composed material representing the three primary progenitors of *M. domestica*. Accessions belonging to a group of tiny-fruited species (*M. baccata*, *M. toringo*, *M. prunifolia*, and *M. spectabilis*) and their hybrids (among themselves, but not with the three primary progenitor species) were collectively designated as the “exotic *Malus*” group (*n* = 44). While the species and hybrids within this group were genotypically and phenotypically distinct, they were grouped together because they presented a relatively higher incidence of null alleles and similar genotypic profiles (but quite different to *M*. *domestica* cultivars and accessions of the primary progenitor species) when analyzed on the 20 K SNP array. These issues were likely due to ascertainment bias inherent in the array design. The primary progenitor group and the exotic *Malus* group included in the panel of wild accessions (hereafter referred to as the “wild panel”) were used for assigning AIMs (Table S1). Wild species accessions previously identified as having clear *M. domestica *admixture [15] were excluded from this panel. For accessions in known, ordered parent–offspring or parent-parent–offspring trio relationships, only the parent(s) were retained. In two cases, previously imputed* M. sylvestris *parents [12] were included, and their full-sib offspring were excluded. The composition of the wild panel was subsequently updated following application of the initial version of the ancestry-informative allele library to a large set of* Malus *data [26], as the species attributions of some of the individuals in that dataset were determined only during the present study.

A panel of 30 diverse cultivars was chosen to represent *M. domestica *via principal component analysis (PCA) and STRUCTURE analysis (Table S1). This size was chosen to match the smallest species sample size in the wild panel, ensuring more equal accession representation of each panel. Individuals in this panel were chosen to be unrelated or only distantly related to each other, as determined by an analysis of summed potential lengths of shared haplotypes (SPLoSH) (as previously described [33]). The chosen cultivars also met one or more of the following criteria: being key pedigree ancestors at the terminal ends of pedigrees, being especially culturally important cultivars, and/or having been identified as particularly genotypically unique [14, 26, 28]. Cultivars known to be derived from* M. baccata* or other exotic *Malus* species (for example, cultivars descended from *M. floribunda* 821 [38]) were excluded from this panel.

Some individuals of the *M. domestica* panel, along with additional *Malus* cultivars and accessions, were used to validate and refine the species-specific allele library developed in this study. These data were shared by various contributors (e.g., [27, 28]) or generated for an ongoing large-scale pedigree reconstruction project [26], some of which was already published. Additional cultivars and germplasm accessions were also included to demonstrate species clarification and to identify and quantify admixture. Twenty-eight accessions from the United States Department of Agriculture (USDA) National Plant Germplasm System (NPGS) Apple Collection in Geneva, New York, which were previously characterized for the proportion of their genomes attributable to* M. sieversii* and *M. sylvestris* [3], were also included for comparison. All germplasm individuals used are listed in Table S1.* Malus* UNiQue genotype codes (MUNQ) [28, 39, 40] were used to keep track of the identities of duplicate genotypic profiles.

All accessions were genotyped on the Illumina Infinium® 20 K SNP array [24] or their genotypes were imputed from 20 K SNP array data of their offspring and, where available, their parent(s) following previously established methods [33, 35]. All SNP data were curated as described previously [29, 41]. Briefly, individuals with low-quality genotypic data were removed, SNPs with unreliable genotype calls were excluded, and all Mendelian-inconsistent errors and most Mendelian-consistent errors were resolved through inspection of cluster plot data of pedigreed individuals. Data previously curated in other studies were further improved during this study as additional data became available since those studies had been published. A total of 10,321 high-quality SNPs previously used to identify* M. domestica* admixture in wild *Malus* [15] in the NPGS Apple Collection were considered in this study. An updated [41] version of the iGL genetic map [32] was used to estimate the centiMorgan (cM) positions of each SNP. The length of this map was 1,267 cM.

Phased SNP genotypic data were generated through graphical genotyping and SHAPEIT5 [42]. For individuals with at least five genotyped offspring or with one or both parents available, graphical genotyping was performed via a Microsoft Excel tool using the same logic applied in the phasing and imputation of “Unknown Founder 1” as previously described [33]. Briefly, genotypic data from offspring and, when available, their parent(s) were used to graphically reconstruct each parental haplotype per chromosome. All other individuals were phased via SHAPEIT5 [42] with the phase_common tool and the following settings: pbwt-window = 10, pbwt-depth = 25, –hmm-ne 2000, mcmc-iterations = 10b,1p,1b,1p,1b,1p,1b,1p,1b,1p,1000m. These settings were determined to produce phased data that were more consistent with phased data previously generated through graphical genotyping (from Volk et al. [15]). Individuals phased via graphical genotyping were also included in SHAPEIT5 to improve phasing. However, for the wild panel, each species/species group was phased individually for use in generation of the species-specific haploblock allele library to avoid biases from uneven group representation that could influence haploblock allele identities. SHAPEIT5 requires a minimum population size of 50 for phasing; however, such sample sizes were unavailable for the* M. orientalis* and exotic *Malus* groups in this study. Therefore, each individual in these two groups was included twice. The resulting phased haplotype data were expected to be sufficiently accurate across the tightly linked SNPs within haploblocks to enable identification of haploblock alleles specific to these groups. The imputed genotypic data automatically generated by SHAPEIT5 were reverted to missing values to prevent potential downstream effects of erroneous imputation.

### Construction of the species-specific SNP allele library

The wild panel was used to identify species-specific SNP alleles, forming the first panel of AIMs. To ensure accurate species specificity, the wild panel was further refined to exclude misclassified accessions, hybrids, and individuals with clear admixture, following the methods of Volk et al. [15]. Briefly, PCA was performed via prcomp in R [43] to demonstrate separation among species and to clarify the species/species group assignment of each wild accession. PCA was conducted via a matrix of SPLoSH [33] values instead of raw SNP data to incorporate genetic length information and minimize the effects of unequal SNP informativeness. SPLoSH values were generated using a minimum length threshold of 5 cM. Individuals that clustered distinctly between, rather than within, the main species clusters were considered hybrids or admixed and were excluded from the identification of species-specific alleles.

Using the updated wild panel, species-specific SNP alleles were identified across the set of 10,321 SNPs. Alleles were considered species-specific if their frequency was ≥ 10% in one species/species group and if they were detected no more than twice among all the alleles in each of the other species/species groups. The reasons to define an allele as species-specific even when it was detected rarely in other species/species groups were to allow for the possibility of rare genotyping errors and because low levels of admixture were assumed, and in some cases known, to exist for some individuals. A stricter definition of species-specificity in the presence of admixture would likely reduce the detection of true species-specific SNP alleles across genomic regions affected by admixture. A 10% threshold for inclusion was chosen, rather than a lower threshold (e.g., 5%), to provide conservative attribution given the small population sizes of *M. orientalis* and exotic *Malus* in the wild panel. During the analysis, very few alleles were found to be specific solely to *M. sieversii* or *M. orientalis* because the two species appear very closely related. Consequently, alleles specific to these two species together were also calculated.

Species-specific SNP alleles identified in the previous analysis were used to further refine the wild panel for species-specific SNP allele detection. Using this initial set of alleles, wild panel individuals with outlier totals (> 100) of species-specific SNP alleles from species/species groups to which they did not belong were excluded. Although low levels of admixture were subsequently detected for many of the remaining individuals, they were retained in the wild panel because the sample size would have been too small had they been excluded. Alleles with unexpected ancestry in individuals occurring singly or only in pairs were evaluated via cluster plot data to determine if the call was because of an errant cluster position and, if so, the call was corrected. To prevent genomic regions with evidence of admixture from influencing downstream analyses, regions in individuals containing at least one species-specific SNP allele from species/species groups to which the individuals did not belong were masked (i.e., set to missing data). The boundaries of these masked genomic regions were defined by SNPs homozygous for species-specific SNP alleles from the species/species group to which the individual belonged. For the exotic *Malus* group, the boundaries were also defined by SNPs homozygous for null alleles, as homozygous null calls were far more common in the exotic *Malus* than in the other *Malus* species genotyped on the 20 K array.

The library of species-specific SNP alleles was finalized using the masked wild panel data with a 10% presence threshold as before, but with the added stringency that each species-specific allele must also not be present in the masked haplotype data in each of the other species/species groups individually. A list of additional SNP alleles specific only to *M. domestica* was also identified. These *M. domestica*-specific alleles were not observed in the masked wild panel but were observed in at least 10% of all the alleles of the 30 *M. domestica* individuals used for PCA and STRUCTURE analyses.

### Construction of the species-specific haploblock allele library

Following the development of the species-specific SNP allele library, a corresponding library of species-specific haploblock alleles, the second panel of AIMs, was generated. Haploblocks were first defined as regions containing a minimum of three SNPs, less than 0.2 cM, and spanning less than 300,000 nucleotides using previously determined physical coordinates [41] on the GDDH13v1.1 whole-genome sequence [30]. SNPs with high frequencies of null alleles, missing data, or problematic clustering were excluded from haploblocks.

Using the phased data from SHAPEIT5, haplotypes indicative of admixture were masked. The boundaries of the masked regions extended to where the expected species-specific alleles were present. For the exotic *Malus* group, the boundaries also included SNPs that were homozygous null.

The species-specific haploblock allele library was constructed via the haploblock definitions and masked phased data from the wild panel. The frequencies of each haploblock allele within each species/species group were calculated via a custom R [43] script. Species-specific haploblock alleles were then defined as those comprising at least 10% of the observed haploblock alleles of a species/species group but not being observed in the other species/species groups.

The species-specific SNP and haploblock allele libraries were then refined by comparing phased data of highly curated individuals to identify and exclude alleles that did not appear to be as species-specific as the data from the wild panel had indicated. Specifically, exotic *Malus* alleles occurring singly (i.e., not within a group of linked alleles unbroken by alleles specific to the primary progenitors of *M. domestica*) in multiple unrelated *M. domestica* cultivars otherwise lacking multiple exotic *Malus*-specific alleles were excluded from the set of exotic *Malus*-specific alleles, as these were considered false positives. Similarly, cultivars known or later confirmed to have a large exotic *Malus* component were inspected to determine whether any non-exotic *Malus*-specific alleles were found singly and closely flanked (less than 1 cM on either side) by haplotypes otherwise characterized by the presence of exotic *Malus*-specific alleles in at least five individuals. Any such alleles were excluded from the set of species-specific alleles.

### Estimation of ancestral species composition with species-specific SNP and haploblock allele libraries

The ancestral species compositions of *M. domestica* cultivars and germplasm accessions with unknown or ambiguous species attributions were estimated via the application of two panels of AIMs (the species-specific SNP and haploblock allele libraries). For each individual, the presence of species-specific alleles was assessed at each AIM. Genomic regions lacking species attribution but flanked by alleles specific to the same species or species group were considered attributable to that species or species group if the region spanned less than 5 cM. The 5 cM threshold was removed for regions flanked by exotic *Malus*-specific alleles for two reasons. First, exotic *Malus*-specific alleles were comparatively rare. Second, if extended regions flanked by exotic *Malus*-specific SNPs were actually descended from either *M. sieversii*/*M. orientalis* or *M. sylvestris*, then there would typically be alleles specific to those species present because they are more prevalent. Regions smaller than 5 cM flanked by one allele specific to both *M. sieversii* and *M. orientalis* (i.e., to both species) and another allele specific to only *M. sieversii* or *M. orientalis* (i.e., specific to only one of the two species) were conservatively attributed as being specific to *M. sieversii* and *M. orientalis*(i.e., to both species). Once species origins were assigned where possible, the lengths of attributed regions were summed (in cM) for each homolog within each genotypic profile. Attributed genomic regions were visualized for some example accessions via circular schematic karyograms via a custom R [43] script adjusted from Vanderzande et al. [44] and the R package ggplot2 [45]. Relative ancestry attributions were compared to those reported in Sun et al. [3].

For accessions suspected of admixture that were not included in the wild panel, ancestral proportions were assigned via the same AIM-based method. Wild accessions estimated to have 1–5% admixture were labeled “with minor admixture”; those with 5–10% admixture were labeled “with admixture”; and those with 10–20% admixture were labeled “with substantial admixture.” Accessions originally recorded as wild *M. sieversii*, *M. sylvestris*, or *M. orientalis* but found to have more than 20% admixture from any of these species were classified as *M. domestica*. An exception to these methods was made for ten wild accessions that had more than 10 exotic *Malus*-specific SNP alleles and at least 100 SNP alleles specific to the three primary progenitors of *M. domestica*. These were designated “Hybrid *Malus*”.

### STRUCTURE analyses

Relative ancestral species attributions per individual and per SNP were also assessed via STRUCTURE software [46] and compared with those derived from the species-specific allele libraries. The linkage model was used in STRUCTURE to avoid “admixture linkage disequilibrium” [46]. The linkage model considers alleles that are linked and from the same population. This model achieves more accurate estimates of the ancestry vector [47, 48]. Prior to analysis, null alleles were set to missing data, as their inclusion has been shown to distort ancestry estimates in STRUCTURE analyses [49]. The final STRUCTURE run used 3000 Markov chain Monte Carlo iterations with a 10% burn-in period, with 10 iterations for each K value from 1 to 4. STRUCTURE analyses were performed on eight germplasm sets. The first set included all individuals used in PCA. The results of this analysis, at K = 3, were used to compare ancestry attributions to those made via the species-specific allele library. The second set included only the wild panel. The results of this analysis, also at K = 3, were used to compare allele frequencies per K group with those observed in the masked data from the wild panel for the exotic *Malus* group, *M. sieversii* and *M. orientalis* together, and *M. sylvestris*. The third set included only the three primary *M. domestica* progenitors and was compared with the PCA results. The fourth analysis included only *M. sieversii* and *M. orientalis *and was used to determine how well STRUCTURE could distinguish between these two species. The last four analyses were conducted using only accessions within each species, or species group, individually. The optimal K value was determined on the basis of the LnP(D) values reaching a plateau [46] and Evanno ΔK statistics [50] via STRUCTURESELECTER software [51]. The final results were consolidated via CLUMPP v1.1.2 [52].

## Results

### Population structure of the test set

**Fig. 2:**
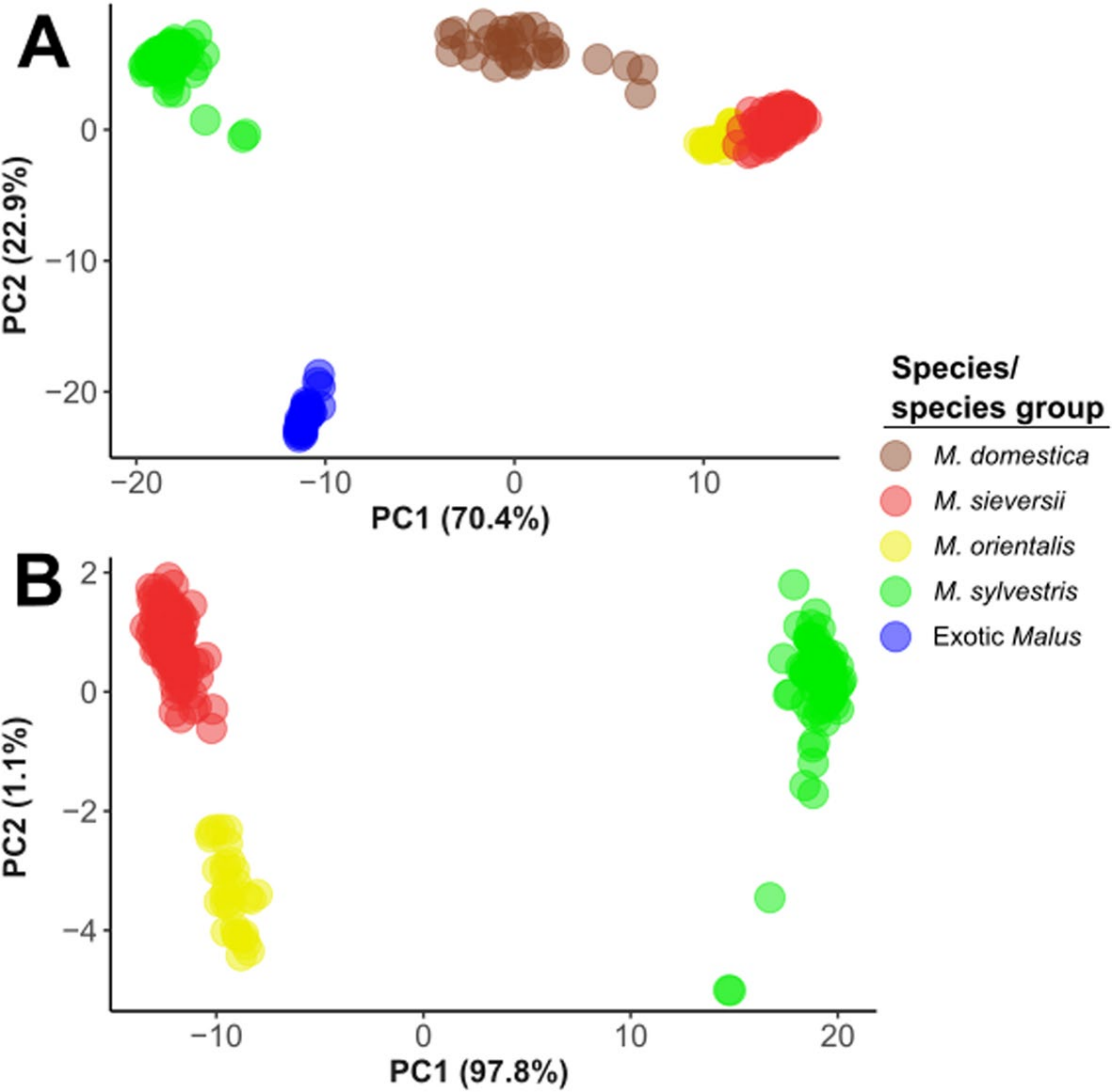
Principal component analysis (PCA) of SNP genotypic data for *Malus* species and cultivars. **A** PCA including *M. domestica*, *M. sieversii*, *M. orientalis*, *M. sylvestris*, and exotic *Malus*. Outlier *M. domestica* cultivars are Api, Fyriki, Kantil Sinap, and M.9. **B** PCA excluding *M. domestica* and exotic *Malus* accessions. Outlier *M. sylvestris* individuals are the Italian accessions M_sylvestris_2, M_sylvestris_5, and M_sylvestris_10. Input data were based on summed potential lengths of shared haplotypes between each pair of individuals, using 5 cM as a threshold. Overlapping datapoints are represented by a darker shade of color

The final wild panel of effectively pure species representatives consisted of 110 *M. sieversii*, 85 *M. sylvestris*, 30 *M. orientalis*, and 31 exotic *Malus* individuals (Table S1). Each species/species group was differentiated via PCA (Fig. 2). The first principal component (capturing 70% of the genotypic variation) distinguished *M. sylvestris* from an overlapping *M. sieversii* and *M. orientalis* cluster, with the *M. domestica* cultivars clustered between them and the exotic *Malus* group clustering between the *M. domestica* cultivar and *M. sylvestris* clusters (Fig. 2A). The cultivars Api, Fyriki, Kantil Sinap, and M.9 were outliers and were positioned much closer to the *M. sieversii*-*M. orientalis* cluster than all other *M. domestica* individuals. The second principal component (23% variation captured) separated the exotic *Malus* group from the other *Malus* species. A second PCA excluding *M. domestica* and exotic *Malus* accessions (Fig. 2B) revealed that all *M. orientalis* accessions were separated from the *M. sieversii* accessions along the second principal component, although this axis explained only 1% of the total variance compared with the 98% captured by the first principal component, which separated *M. sylvestris* from *M. sieversii* and *M. orientalis*. Three Italian *M. sylvestris* accessions clustered separately from the main *M. sylvestris* cluster in both PCAs (Fig. 2).

**Fig. 3:**
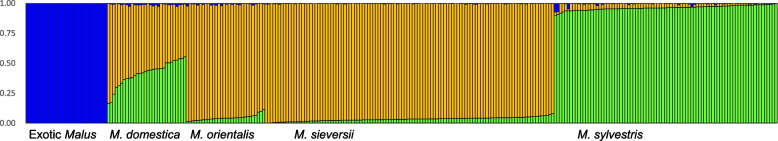
Ancestry probability attributions (represented as vertical bars) using the optimal grouping of K = 3 (represented by the three colors) from the results of STRUCTURE analysis of a panel of 30 *M. domestica* cultivars, 110 *M. sieversii* accessions, 30 *M. orientalis* accessions, 85 *M. sylvestris* accessions, and 31 exotic *Malus* accessions

The first STRUCTURE analysis identified K = 3 as the optimal K value (Figure S1) and separated *M. sylvestris* and exotic *Malus* into their respective groups, however *M. sieversii* and *M. orientalis* grouped together (Fig. 3). The ancestral composition of the *M. domestica* cultivars averaged 42% from *M. sylvestris*, 57% from *M. sieversii*/*M. orientalis*, and 1% from exotic *Malus*. From this analytical approach, exotic *Malus* was also detected at an average level of 0.2% in *M. sieversii*, 0.7% in *M. orientalis*, and 0.5% in *M. sylvestris*. Regardless of the species subsets included in STRUCTURE, *M. sieversii* and *M. orientalis* were not separated.

### Species-specific SNP and haploblock allele libraries

Large numbers of AIMs were identified (Table 1). Across the four groups of the wild *Malus* panel included in the study, 4426 species-specific SNP alleles (listed in Table S2) and 4357 species-specific haploblock alleles (listed in Table S3) were identified across the 10,321 SNPs and 1395 haploblocks, respectively. Few alleles were identified to be specific only to *M. sieversii* or *M. orientalis*, although many were specific to both species combined. There were fewer exotic *Malus*-specific alleles than those specific to *M. sylvestris* or *M. sieversii* and *M. orientalis* combined (Table 1). The exotic *Malus*-specific haploblock alleles helped to fill in areas lacking exotic *Malus*-specific SNP alleles. Despite the high frequency of null alleles, the exotic *Malus* accessions presented distinct genome-wide haplotypes when genotyped with the 20 K SNP array, which was observed as a high degree of homozygosity, often for the same alleles among unrelated exotic species. Consequently, the 20 K SNP array genotypic profiles of exotic *Malus* species were poorly distinguished from each other but readily stood out from those of *M. domestica* and its main progenitor species.

**Table 1:** Total species-specific SNP and haploblock alleles identified for each species/species group across a set of 10,321 SNPs and 1095 haploblocks included in this study

Species or species group	Species-specific SNP alleles	Species-specific haploblock alleles	Species-specific haploblock alleles that lacked species-specific SNP alleles
*M. sieversii*	38	132	40
*M. orientalis*	15	85	20
*M. sieversii* or *M. orientalis*	2146	1751	376
*M. sylvestris*	1883	1688	399
exotic *Malus*	221	701	581
*M. domestica*	123	NA	NA
Total	4426	4015	1190

### Ancestral species composition and admixture estimations of wild accessions

SNP allele frequencies per SNP for each of the three STRUCTURE-defined group (*M. sylvestris*, *M. orientalis* and/or *M. sieversii*, and exotic *Malus*) were highly correlated with those in the species-specific allele library, with some differences (Figure S2, Table S4). An example of a difference: For the 20 K SNP index number 8848, the *A* allele was assigned exclusively to K1 (*M. sylvestris*) in the STRUCTURE results, whereas in the allele library it occurred at a low frequency in both *M. sieversii* and *M. sylvestris*. Although certain SNPs showed notable frequency deviations between STRUCTURE and the masked data used to create the species-specific SNP allele library (Table S4), most of these were detected in the exotic *Malus* group and involved SNPs with frequent null alleles. The other notable exception was that, compared with the species-specific allele library, STRUCTURE attributed much greater species specificity for *M. sieversii* and *M. orientalis* to a set of 8 adjacent SNPs over a 9-SNP region on chromosome 10 (35.09–35.11 cM).

**Fig. 4:**
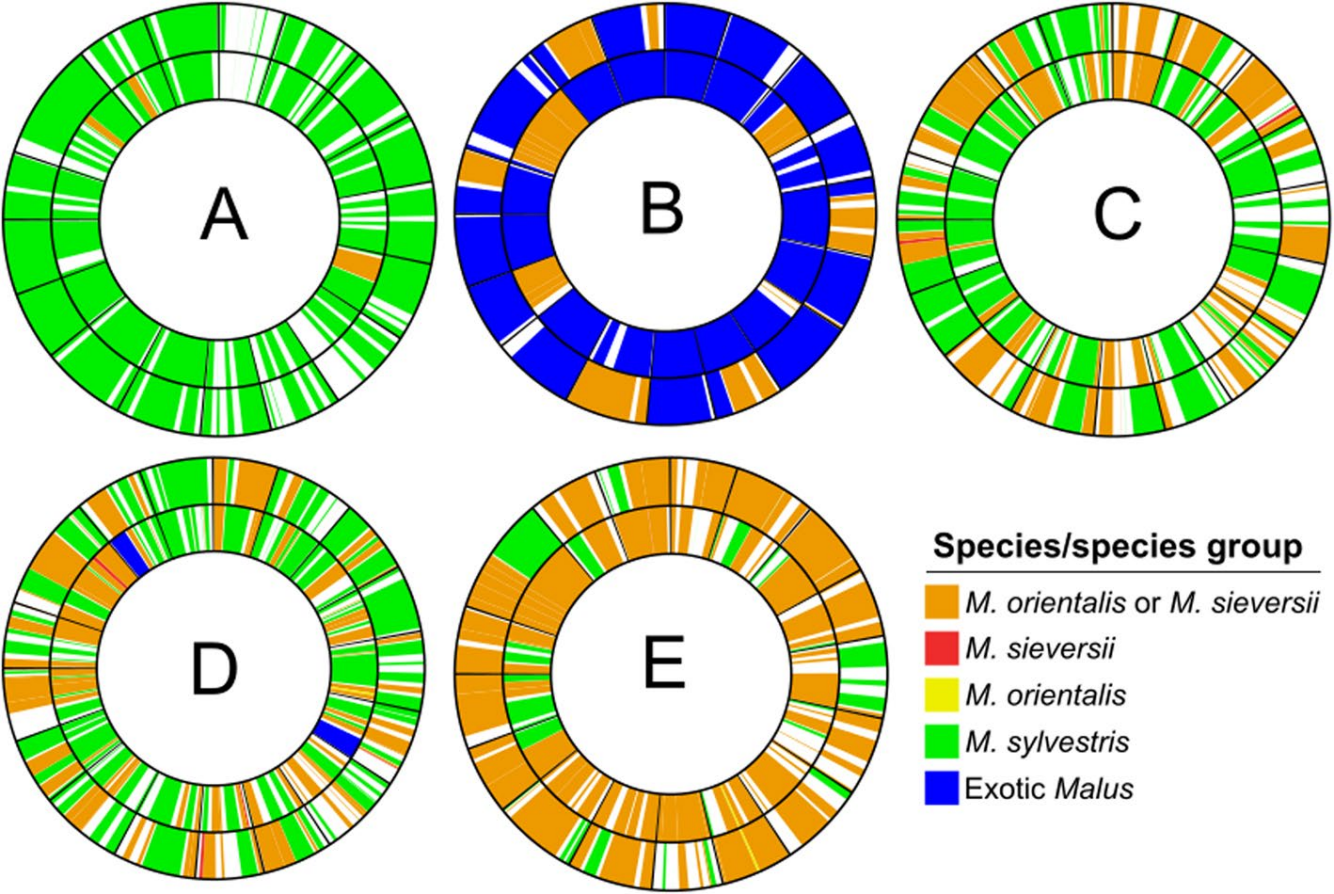
Circular schematic karyograms displaying ancestral species compositions of example *Malus* accessions and cultivars. (**A**) PI 633825 (a German *M. sylvestris* accession in the USDA collection), (**B**) *M.* × *floribunda* 821 (a tiny-fruited hybrid *Malus* accession, previously reported as exotic, used in disease resistance breeding), (**C**) ‘Blanc Mollet’ (a French cider cultivar), (**D**) ‘Honeycrisp’ (an important modern dessert cultivar), and (E) ‘Api’ (an ancient French dessert cultivar). Chromosomes are arranged clockwise from the 12 o’clock position, beginning with chromosome 1 and continuing through chromosome 17. Both homologs of each chromosome are shown. For ‘Honeycrisp’, the contribution from its mother is represented by the inner ring, whereas for the other individuals, the parental attribution per chromosome was unknown and thus randomly assigned

Many accessions in the wild panel were determined to have low levels of admixture (Table S5). Most *M. orientalis* accessions presented some admixture from *M. sylvestris*, and most *M. sylvestris* accessions presented some admixture from *M. sieversii* and/or *M. orientalis.* All the accessions included in the final panel presented less than 3.7% admixture (via total admixture cM over the total cM attributed), and only four *M. orientalis* and 31 *M. sylvestris* accession presented more than 2% admixture. This low level of detected admixture was often present in the form of a few extended haplotypes (> 5 cM) containing numerous species-specific alleles from the admixture source. For example, admixture in *M. sylvestris* accession PI 633825 was detected almost exclusively from three extended haplotypes (Fig. 4A). These segments, on chromosomes 6, 15, and 16, were at least 29.5 cM, 10.3 cM, and 11.4 cM in length and contained 27, 24, and 16 *M. sieversii* or *M. orientalis*-specific alleles, respectively. Eighteen *M. sylvestris* accessions, mostly from Dutch *M. sylvestris* accessions, were determined to have likely admixture at various portions of the top of chromosome 1. This could have been due to population-specific admixture or a lack of SNPs with alleles that could easily distinguish between the species in this area. Compared with the other accessions of *M. sylvestris*, the three Italian *M. sylvestris* accessions all presented either no or very low levels of admixture but lower levels of *M. sylvestris*-specific alleles. Many accessions originally labeled as *M. sieversii* presented admixture, but there were enough pure or nearly pure *M. sieversii* accessions that those with clear admixture were able to be excluded from the wild panel. Species assignments based on STRUCTURE and the species-specific allele libraries were consistent, although STRUCTURE generally produced higher admixture estimates than did the allele library, particularly for *M. sieversii* (Figure S3, Table S5).

Species attributions of wild accessions were clarified or confirmed for several cases, while unexpected admixture was detected in others (Table S5). The important breeding accession ‘Russian Seedling’ was identified as being > 99% *M. sieversii*/M*. orientalis* and clearly clustered with *M. orientalis* and not *M. sieversii* in the PCA. Similarly, ‘Niedzwetzkyana’ was determined to be pure *M. sieversii*. The accession *M.* × *floribunda* 821 was estimated to constitute 20.2% *M. sieversii*/*M. orientalis* and only 79.8% exotic *Malus* (Fig. 4B). Several accessions originally labeled *M. prunifolia* (PI 613847, PI 687698, and ‘Nagano’) were identified as hybrids, combining ancestry from *M. sieversii* (with a possible minor *M. orientalis* contribution) and exotic *Malus*. PI 687698 also had a small *M. sylvestris* component. In contrast, *M. prunifolia* accession PI 588994 was identified as a purely exotic *Malus*. Comparable mixed ancestry patterns were observed in the *M. baccata* and *M. toringo* accessions, with some showing substantial *M. sylvestris* admixture (Table S5).

### Ancestral species composition of *M. domestica* cultivars

All the *M. domestica* cultivars were identified as admixtures of *M. sieversii* and/or *M. orientalis* and *M. sylvestris*, with some having a small exotic *Malus* component. An average of 69.8% (63.1–76.5%) of each genotypic profile among the examined cultivars recorded originally as *M. domestica* was attributed to an ancestral species origin (Table S5). Because 23.5–36.9% of the genomic regions were not able to be confidently attributed to any species, it was not fully conclusive as to whether *M. sieversii* and *M. orientalis* composed more of the genotypic profiles of *M. domestica* than did *M. sylvestris*. However, considering the proportions of genotypic profiles attributable to the progenitor species, the ancestral composition clearly differed among many of the cultivars (Fig. 4, Table S5). Ten cultivars (‘Blanc Mollet’ (Fig. 4C), ‘Dabinett’, ‘Duchess of Oldenburg’, ‘Gala’, ‘Golden Delicious’, ‘Greensleeves’, ‘Honeycrisp’ (Fig. 4D), ‘Hvid Vinter Pigeon’, ‘Roter Jungfernapfel’, and ‘Smith’s Cider’ had more than 50% of their genotypic profiles attributable to *M. sylvestris*, whereas all other 39 cultivars tested did not. ‘Blanc Mollet’ and ‘Honeycrisp’ had the highest proportions of their genotypic profiles attributed to *M. sylvestris* (59% and 56%, respectively). The lowest levels of *M. sylvestris* admixture (13.9–28.1%) were detected in the four cultivars previously noted as being outliers in PCA positioned towards the *M. sieversii*/*M. orientalis* cluster (Fig. 2), ‘Api’ (Fig. 4E), ‘Fyriki’, ‘Kantil Sinap’, and ‘M.9’, as well as the rootstock cultivars Budagovsky 54–118 and Budagovsky 9. Correspondingly, the highest proportions of *M. sieversii*/*M. orientalis* in cultivars were detected in ‘Api’ (85.1%) and ‘Kantil Sinap’ (83.9%).

The precise extent of ancestral contributions of *M. sieversii* versus *M. orientalis* to the genotypic profiles of *M. domestica* cultivars could not be determined, as relatively few alleles that were specific to either species alone were identified. Nevertheless, every *M. domestica* cultivar had some haplotypes attributed to *M. orientalis* and to *M. sieversii* individually. Some *M. domestica* cultivars presented clear exotic *Malus* admixture, including ‘Novosibirski Sweet’ (32%), the rootstock cultivar Budagovsky 54–118 (18.8%), ‘Liberty’ (4.1%, known to have exotic *Malus* from *M.* × *floribunda* 821 in its recent history), and ‘Honeycrisp’ (2.6%, Fig. 4D). ‘Dolgo’, a multi-use cultivar known to be a hybrid crab apple, was estimated to be 40.5% exotic *Malus*.

Modern cultivars tended to have greater fragmentation of ancestral haplotypes from their wild progenitors compared to older cultivars (column Q in Table S5). For example, the modern cultivar Honeycrisp (Fig. 4D) presented greater fragmentation of species-attributed haplotypes than did the old cider cultivar Blanc Mollet (Fig. 4C) and the ancient French cultivar Api (Fig. 4E). However, this trend was not consistent, as some older cultivars also presented high levels of fragmentation, specifically ‘Hawthornden’ (first recorded in 1780 [53]) and ‘Smith’s Cider’ (circa 1800 [54]).

Higher numbers of “*M. domestica*-specific” alleles were generally observed in modern cultivars and those closely related to them, particularly ‘Golden Delicious’ (Table S5). When the genomic positions of these alleles were compared to the proximity and presence of species-specific SNP alleles from other species, many alleles were present in haplotypes that contained closely flanking alleles specific to *M. sieversii*/*M. orientalis* or to *M. sylvestris*. 

The relative ancestry attributions for cultivars obtained from the species-specific allele library were highly consistent with the relative ancestry attributions from STRUCTURE (Figure S4). However, STRUCTURE consistently assigned a small exotic *Malus *component to all cultivars, whereas the allele library approach did not detect clear exotic ancestry in the same individuals. Comparisons with the results of Sun et al. [3] also showed strong correlations. Ancestry proportions attributed to* M. sieversii*/*M. orientalis* together and to *M. sylvestris* via the species-specific allele library were highly correlated with the relative ancestry attributions of *M. sieversii* (and/or *M. orientalis *in Sun et al. [3]) (R^2^ = 0.69) and *M. sylvestris* (R^2^ = 0.75), respectively (detailed in Table S6). Two cultivars, Novosibirski Sweet and M.9, were clear outliers that reduced the correlation strength. When those two accessions were excluded, the R^2^ values were 0.89 (*M. sieversii*) and 0.96 (*M. sylvestris*). A slightly higher average proportion (75.4%) of cultivar genomes were attributable to the species-specific ancestries in Sun et al. [3] compared with 69.8% from the species-specific allele library in the present study. Sun et al. [3] did not include exotic* Malus* in their model, so direct comparisons for that ancestry component were not possible.

## Discussion

The identification of AIMs and species-specific alleles at these AIMs in this study enabled unprecedented insight into the ancestral composition of *M. domestica*. While AIMs have been used extensively in human genetics and sometimes in plant species (e.g., in grape–*Vitis*spp. [55]), this is the first study in *Malus* to identify and use AIMs for estimating ancestry and admixture levels. Admixture among *Malus* species has been previously estimated with various statistical analyses (e.g., [2, 3, 14, 56, 57]), but this study is the first to identify the ancestral origin of each chromosomal segment while accounting for admixture in representative species accessions. The novel methods used for*Malus* generated results that were mostly consistent with the more commonly used population genetics tool STRUCTURE and with the results of Sun et al. [3]. The species-specific SNP and haploblock allele libraries developed in this study could be used to probe the relative ancestral contribution of the primary* M. domestica* progenitors of any *Malus* individual if that individual is genotyped with some or all the AIMs identified. This genotyping could be accomplished using additional SNP array data, but also DNA sequence data, as long as allele calling between the target SNPs is concordant.

### Clarification of the ancestry of wild *Malus* accessions

The ancestral composition of many wild accessions was clarified. ‘Niedzwetzkyana’ (PI 589857 in the NPGS collection) was confirmed as being purely *M. sieversii*. This attribution was previously assumed or speculated by some [58, 59] and disputed by others [60, 61]. While some groups consider Niedzwetzkyana to be a group of individuals, the results of this study apply to the genotypic profile represented by clonal accessions commonly held in many germplasm collections (e.g., PI 589857), which is the pedigree source of red flesh in many cultivars, e.g., a parent of ‘Geneva’ [59]. ‘Russian Seedling’, previously reported as* M. pumila* [62] and* M. domestica* (USDA GRIN accession PI 588849), clearly clustered with *M. orientalis* in the PCA. Both ‘Niedzwetzkyana’ and ‘Russian Seedling’ are relevant to current apple breeding efforts as sources of different apple scab (*Venturia inaequalis*) resistance alleles (*Rvi3* [63] in ‘Geneva’ and*Rvi2* and *Rvi4* [64] in ‘Russian Seedling’). Clarification of the latter source highlights the importance of* M. orientalis*, which has not previously been reported as an explicit focus of targeted breeding for scab resistance, although the species has previously been reported as harboring disease resistance [65].

Admixture was previously known to be common among the primary progenitors of *M. domestica *in the formation of the crop [15, 66, 67], but this study was able to detect small levels of introgression not previously documented in other studies that included the same accessions and had otherwise considered them to be pure species representatives (e.g., PI 633801, PI 650955, and PI 650983 from the NPGS were previously listed as not having admixture [15]). While some of this detected introgression might be due to limitations of the analysis (discussed later), much was detected in the form of multi-cM haplotypes composed of numerous species-specific alleles, providing confidence in the presence of admixed regions and suggesting generationally recent admixture. For example, the* M. sylvestris *accession PI 633825, previously used to create a whole-genome sequence [3] to represent this species, was detected in the present study to be 2.8%* M. sieversii* and/or *M. orientalis* (Fig. 4A, Table S5). This unexpected admixture was almost exclusively present in three haplotypes that were each longer than 10 cM and contained close to or more than one *M. sieversii* and/or *M. orientalis*-specific allele per cM. Accounting for such admixture might inform subsequent studies on the ancestral origins of the alleles of interest, the domestication history of *M. domestica*, and the evolutionary history of *Malus*. Using this study’s approach and results to verify the species purity of the researched accessions would also help future studies that seek to establish species-specific genomic resources such as whole-genome sequences.

Admixture was also common among the exotic *Malus* accessions examined, with some taxonomic inconsistencies compared with previous literature. Many supposedly “*M. baccata*” accessions were detected here to have substantial admixture (Table S5), including ‘Hansen’s baccata #2’, the source of the scab resistance allele *Rvi12* [68]. This finding corroborates a previous report of admixture in* M. baccata* accessions in which SSR data were used [57]. Few accessions of* M. prunifolia* and even fewer of *M. toringo* were included in the present study, but there was inconsistency among species compositions for these species, with some accessions being purely exotic *Malus* while others had clear, and often substantial, admixture. The finding that the *M. prunifolia *accessions were not a clearly defined singular species was consistent with reports of previous studies [9, 57, 69]. *M.* × *floribunda *821, the source of the apple scab resistance allele Rvi6 [38], was estimated here to be 20.2%* M. sieversii* and/or *M. orientalis*, although *M.* × *floribunda* is reported only as a hybrid between the exotic crab apple species *M. baccata* and *M. toringo* [70]. The results of this study and those of previous studies [15, 57, 66, 67, 69] collectively indicate that* Malus* taxonomy needs clarification, as many reported *Malus* “species” are convoluted mixes of pure wild accessions, hybrids, admixed accessions, and *M. domestica* cultivars. Resolving or annotating these taxonomic inconsistencies would benefit breeders, conservationists, and other scientists.

### Ancestral species composition of *M. domestica* cultivars

The results of this study are in agreement with the prevailing theory that *M. domestica* is an admixture of *M. sieversii* (and/or *M. orientalis*) and *M. sylvestris*, with some cultivars having clear or possible small contributions from exotic *Malus* species. The large contribution of *M. sylvestris* detected is consistent with the growing body of literature [6, 9] reporting the importance of the genetic contribution of this species to domesticated apple.* M. sieversii* was also confirmed as a primary progenitor species of *M. domestica*. This study could not definitively differentiate *M. sieversii* alleles from those of *M. orientalis* evenly across the genome via the set of SNPs included from the Illumina Infinium 20 K SNP array. However, *M. orientalis* was also determined to be a significant contributor to *M. domestica *cultivars because species-specific alleles from both species were identified in every cultivar included in the study (including Russian-derived ‘Alexander’ and ‘Duchess of Oldenburg’). This latter finding contradicts a hypothesis from a previous study [69], which posited that some cultivars from the former Soviet Union might have developed without introgression with* M. orientalis*. More cultivars and a SNP set with a larger number of genome-wide alleles specific to each progenitor species would enable further examination of this topic.

The results of the only other published study that used SNP data to attribute relative contributions of the primary *M. domestica* progenitors to cultivars, Sun et al. [3], were generally consistent with those of the present study. There were two notable outliers: ‘M.9’ and ‘Novosibirski Sweet’. The inconsistency with ‘Novosibirski Sweet’ might be because the previous study did not include exotic *Malus* ancestry in their model. The importance of including exotic *Malus* was demonstrated by the present study, which detected 17.5% of the genotypic profile of ‘Novosibirski Sweet’ as exotic *Malus*. The inconsistency with ‘M.9’ is less clear but might represent cultivar mislabeling. Another study [8] reported that the same accession of ‘M.9’ had extensive admixture with* M. baccata*, which is inconsistent with the results of offspring of ‘M.9’ (‘M.26’ and ‘M.27’) in that study, as well as with the present study, which revealed no contribution from exotic *Malus *species. While SSR data from GRIN were consistent with the identity of our ‘M.9’ genotypic profile (unpublished comparison to Denancé et al. [40]), this inconsistency could be because of an accession or DNA mix-up. Despite these inconsistencies, the similar results between the present study and Sun et al. [3] supports the ancestry attribution methodology of the present study, despite limitations of the set of SNPs used.

In this study, the answer to the question of “Which progenitor, *M. sieversii* or *M. sylvestris*, contributed the most to the genome of *M. domestica*?” is that it is cultivar specific, with some general trends evident. Most cultivars in the present study presented more of their genotypic profiles attributable to *M. sieversii* (and partly *M. orientalis*) rather than *M. sylvestris*, but ten cultivars presented the opposite results. Among those ten were the cider cultivars Blanc Mollet, Smith’s Cider, and Dabinett (Table S5). Relatively greater *M. sylvestris *ancestry attributable to cider cultivars was reported in one study [14] but not in another [2]. These differences were likely due to the specific cultivars evaluated, as the cider cultivar Bisquet (MUNQ identity code 535) had a lower than average genetic contribution from* M. sylvestris *in the present study. While selection signatures for specific attributes and thus alleles in specific genomic regions from each progenitor have been detected in domesticated apple [9], the relative contributions from each progenitor species are likely also largely a function of cultivar provenance. The importance of provenance for ancestry is evident in the set of individuals with exceptionally higher estimated proportional contributions of* M. sieversii* (and/or *M. orientalis*) to their genomes. Two of these cultivars, Fyriki and Kantil Sinap, are traditional cultivars recorded as originating from southeastern Europe. These cultivars might have less *M. sylvestris* ancestry because they arose on the outer fringe of the native range of *M. sylvestris*, and as traditional cultivars they might be particularly ancient, with less admixture of their *M. sieversii* ancestors with *M. sylvestris* or some admixing with genetically divergent *M. sylvestris *individuals not included in our analysis. This hypothesis regarding more ancient apples might also apply to another outlier, the ancient French cultivar Api. In both the present study and that of Sun et al. [3], ‘Api’ was the cultivar with the highest ancestry proportion attributable to*M. sieversii* (and/or *M. orientalis*) and detected in long haplotypes including spanning several entire chromosomes. The fourth outlier, the rootstock cultivar Budagovsky 9, was recorded as a cross between ‘M.8’ and ‘Red Standard’ (also known as ‘Krasnij Standard’) in a Soviet program beginning in 1938 [71]. While little is known about ‘Red Standard’, at least in English language literature, ‘M.8’ is recorded as an ancient rootstock whose origin might be traced to western Asia [72, 73]. Thus, the age and origin of parent ‘M.8’ could explain why ‘Budagovsky 9’ had such low *M. sylvestris* ancestry. The rootstock cultivar M.9 also had notably lower ancestry attributable to *M. sylvestris*. An attempt was made in the present study to relate haplotype fragmentation to cultivar age, as newer cultivars would be expected to have accumulated more recombination, resulting in greater fragmentation of progenitor species’ haplotypes. While there seemed to be a general trend of greater fragmentation in more recent cultivars, the relatively high fragmentation calculated for some older cultivars suggests that the degree of haplotype fragmentation of progenitor species is not a reliable indicator of the relative age of cultivar origin.

The results of this study clearly revealed that exotic *Malus* admixture is not common in *M. domestica* cultivars (Table S5), excluding cultivars where exotic *Malus *was explicitly incorporated in modern breeding programs (e.g., as in ‘Liberty’ [19]). The identification of exotic* Malus* ancestry in ‘Honeycrisp’ is novel. This ancestry is from its grandparent ‘Frostbite’ (Table S5). The results of a few cultivars with exotic *Malus *species ancestry are consistent with those of Chen et al. [8], although that study, which used STRUCTURE analysis, did not report any exotic *Malus* species ancestry (as *M. baccata* or *M. hupehensis*) in ‘Honeycrisp’. The apparent detection from our STRUCTURE analysis (but not the allele library method) of low levels of exotic *Malus* ancestry in all cultivars is probably a statistical artifact, as the available data did not result in an optimal STRUCTURE analysis. Specifically, the population sizes of the groups were uneven, no corrections were made for SNPs with low minor allele frequencies, and SNP ascertainment bias was inherent in the experimental design because of the *M. domestica*-focused SNP array used. All of these issues have been reported to cause problems in STRUCTURE analyses [74–76].

Overall, the results of progenitor species’ ancestry attributions to cultivars provide a tantalizing glimpse into otherwise murky provenance information available for the origins of *M. domestica* and each cultivar. Higher-resolution ancestry attributions with additional SNPs and an updated AIM library, a species-specific allele library, and a species subpopulation-specific library could provide additional clarity on this topic.

### Limitations of the study

Although this study represents a considerable advancement in ancestry inference in *Malus*, it has several limitations. While the Illumina Infinium 20 K SNP array provided a consistent set of SNPs for creation of the species-specific allele libraries, ascertainment bias and array design were clear limitations. The Illumina Infinium 20 K SNP array used the ‘Golden Delicious’ genome version 2.0 [24], which was improved with subsequent genome publications (e.g., [30, 31, 44]). This reliance resulted in some genomic gaps in SNP coverage. The panel used to choose SNPs on this array included only 16 apple accessions. This panel did not include any accessions of the three primary progenitor species of* M. domestica* and only two accessions with an extensive exotic *Malus* genetic background: *M.* × *micromalus* (named “*M.* × *micromalus* (WUR)” in this study) and F2 26,829–2-2 (reported according to original pedigree records as 50% *M.* × *floribunda* 821). This limited panel likely generated an uneven distribution of species-specific alleles from each species/species group, with some genomic regions particularly lacking such alleles, such as the top of chromosome 1 for *M. sylvestris*. The species-specific haploblock allele library helped overcome these deficiencies, but not completely, as an average of 24.1% of each genotypic profile lacked ancestry attribution even when gaps < 5 cM long that were flanked by species-specific alleles attributable to the same species or species group were filled.

SNP ascertainment bias on SNP arrays has been well documented to influence ancestry inference (e.g., in human genetics [77]) and likely influenced the results of this study. Evidence of SNP ascertainment bias was evident in the relatively low number of exotic* Malus*-specific alleles and the low number of *M. sylvestris*-specific alleles observed in the Italian *M. sylvestris* accessions. Similar to the lack of exotic *Malus* species in the discovery panel, Italian *M. sylvestris* ancestry might not have been directly represented on that panel at all. Another effect of this lack of representation probably contributed to the detection of some isolated haploblock alleles present only in the exotic *Malus* and Italian *M. sylvestris* accessions. These alleles were never in close proximity with others (i.e., not forming longer haplotypes than the individual blocks) in the Italian *M. sylvestris* accessions, suggesting that they were not evidence of true exotic *Malus* admixture. These haploblocks might be composed of only SNPs that are polymorphic among material better represented on the array, meaning that any outgroups, in this case, the exotic *Malus* and Italian *M. sylvestris*, would exhibit the same haploblock allele. This same artifact could have resulted in the presence of individual exotic *Malus*-specific haploblock alleles (of length 0 cM, which is why they didn’t show in Table S5) in some cultivars. Presumably, this artifact also accounted for the large proportion of shared SNP and haploblock alleles among all exotic *Malus* species accessions despite little objective genetic affinity among species within the group.

Another way in which SNP ascertainment bias affected the results was the many SNPs with null alleles in exotic *Malus* accessions. Null alleles were not a problem for the pure exotic *Malus* accessions, as homozygous null calls were clearly separated in genotyping cluster plots. However, for hybrid *Malus* accessions that had large exotic components, particularly those where one parent was likely fully exotic and no offspring were available to use for calling the heterozygous null alleles in the data curation pipeline, many SNPs that appeared homozygous would actually be heterozygous for the null allele. Often, heterozygous null groups partially overlapped with the homozygous groups in the SNP genotyping cluster plots. Therefore, if an individual is determined to have a substantial exotic *Malus* component, SNPs with common null alleles should be excluded from ancestry attribution to avoid instances of false homozygosity that are truly heterozygous null.

Related to the limitation of SNP ascertainment bias is the reliance on a single genetic map. The iGL map [32] represents an approximation of genetic distances among markers but was developed from the earlier iGL map [32], which used 1586 individuals from 21 full-sibling* M. domestica* families. The estimates of genetic distance or even the location of some markers might differ substantially for some of the wild material. However, the published *M. sieversii* and *M. sylvestris* genomes were nearly perfectly collinear with those of *M. domestica*, as were the recently published genomes of *M. baccata* [78] and even the more distant North American species* M. fusca* [79], although the latter presented several small translocations and more inversions than did the other genomes when compared with those of* M. domestica*.

Another limitation was that the species-specific allele library from this study was optimized according to the type of germplasm included in the study. Material from more distant *Malus* species (e.g., *M. fusca* and *M. coronaria*) or hybrids with them would not be expected to be well identified as such using the definitions of species-specificity from this study and because these species perform poorly on the Illumina Infinium 20 K SNP array. Additionally, some of the species-specific alleles identified might not be as specific as they appear from this study because of the species panel sizes and individuals included. The *M. sylvestris* accessions included were mostly from Germany and the Netherlands, with few to no accessions from other *M. sylvestris* populations [80, 81]. There were also relatively few samples of* M. orientalis* and exotic *Malus* species. The use of a larger panel might reveal that some of the species-specific alleles here are actually present in other species or species groups from populations that were not sampled. For the same reasons, some alleles were likely truly specific to a species or species group but not identified as such. The evidence of the latter is that many of the *M. domestica*-specific alleles were often observed very closely flanked by alleles specific to one species or species group.

An improved species-specific SNP and haploblock allele library could be constructed to reduce the limitations of this study. This improvement could be achieved via the use of an updated array (e.g., the Axiom JKI50kMd SNP array first used in Taskuzhina et al. [61]) or sequence data, a larger discovery panel that includes wild accessions, an improved genetic map, alignment to an improved whole-genome sequence or pangenome, and a larger and more diverse wild panel. An improved library would be needed for fine-scale ancestry inference, for example, to better differentiate between contributions from* M. sieversii* and *M. orientalis* and from specific exotic *Malus* species, to identify all and small introgressed haplotypes, and to pinpoint boundaries of introgressed haplotypes and therefore the genes and their alleles included.

## Conclusions

This study significantly advances ancestry determination and admixture detection in *Malus*. The definition and use of species-specific alleles at ancestry-informative markers and subsequent haplotype assignment provided detection of ancestral contributions at the haplotype level while addressing some limitations of other methods. The availability of this library will enable reproducible and consistent admixture detection and ancestry attributions for the ever-growing amount of *Malus* SNP array and sequence data being generated.

All *M. domestica* cultivars were determined a mix of three primary progenitors (*M. sieversii*, *M. sylvestris*, and *M. orientalis*) with a few also having contributions from exotic *Malus* species. Cultivars varied in terms of the relative contributions of each progenitor species, with some evidence that older cultivars and traditional cultivars originating in eastern Europe or Western Asia had less *M. sylvestris* ancestry than modern cultivars and traditional cultivars originating in Western Europe. A larger dataset of such cultivars would be necessary to explore these associations further.

Taxonomic irregularities in wild *Malus* are rampant. Many accessions recorded as belonging to a specific species were determined to instead be hybrids or admixtures. These results should be considered in future evolutionary studies, breeding efforts, and conservation strategies in *Malus*. Subsequent taxonomic studies including ancestry-informative markers should be conducted with a wider array of wild accessions to address these taxonomic issues.

While the species-specific SNP and haploblock allele libraries developed here could be readily applied to other *Malus* SNP genotypic data, the libraries from this study have limitations, mostly stemming from the design of and ascertainment bias inherent in the Illumina Infinium 20 K SNP array. These limitations might need to be overcome to further investigate and clarify the aforementioned taxonomic issues. Additionally, an updated ancestry-informative marker set and species-specific SNP and haploblock allele libraries would be necessary to clarify the contribution of *M. orientalis* to *M. domestica* cultivars.

## Supplementary Information

Supplementary Material 1.

Supplementary Material 2.

Supplementary Material 3.

Supplementary Material 4.

Supplementary Material 5.

Supplementary Material 6.

Supplementary Material 7.

Supplementary Material 8.

Supplementary Material 9.

Supplementary Material 10.

Supplementary Material 11.

## Data Availability

The Illumina Infinium iSCAN array data used in this study have been deposited in the ArrayExpress database under the accession number E-MTAB-16593.

## Abbreviations

AIMS: Ancestry-informative markers
SNP: Single nucleotide polymorphism
SPLoSH: Summed potential lengths of shared haplotypes
