# Supplementary material for: Identifying the contributions of progenitor Malus species to cultivated apple (M. domestica) using 20K SNP array data
Source: BMC Genomics. 2026 Jun 11;27:536. doi: 10.1186/s12864-026-13023-z (PMC13255378; doi:10.1186/s12864-026-13023-z)
Supplement: Supplementary file 1 — Supplementary Material 1. [file 12864_2026_13023_MOESM1_ESM.pdf]

Identifying the contributions of progenitor *Malus* species to cultivated apple (*M. domestica*) using 20K SNP array genotypic data

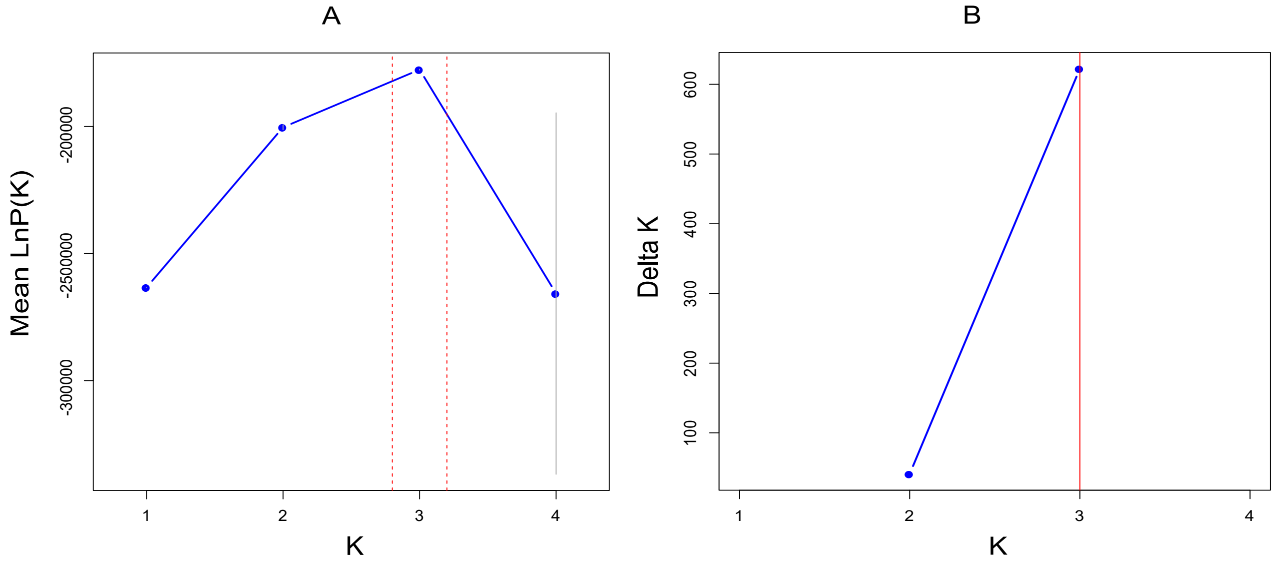

Figure S1: (A) Mean values of likelihood; (B) Delta K of Evanno approach, estimated by means of 10 replications in STRUCTURE to see the optimal K distribution. K was run from K1 to K4.
