# Supplementary material for: Identifying the contributions of progenitor Malus species to cultivated apple (M. domestica) using 20K SNP array data
Source: BMC Genomics. 2026 Jun 11;27:536. doi: 10.1186/s12864-026-13023-z (PMC13255378; doi:10.1186/s12864-026-13023-z)
Supplement: Supplementary file 2 — Supplementary Material 2. [file 12864_2026_13023_MOESM2_ESM.pdf]

Identifying the contributions of progenitor *Malus* species to cultivated apple (*M. domestica*) using 20K SNP array genotypic data

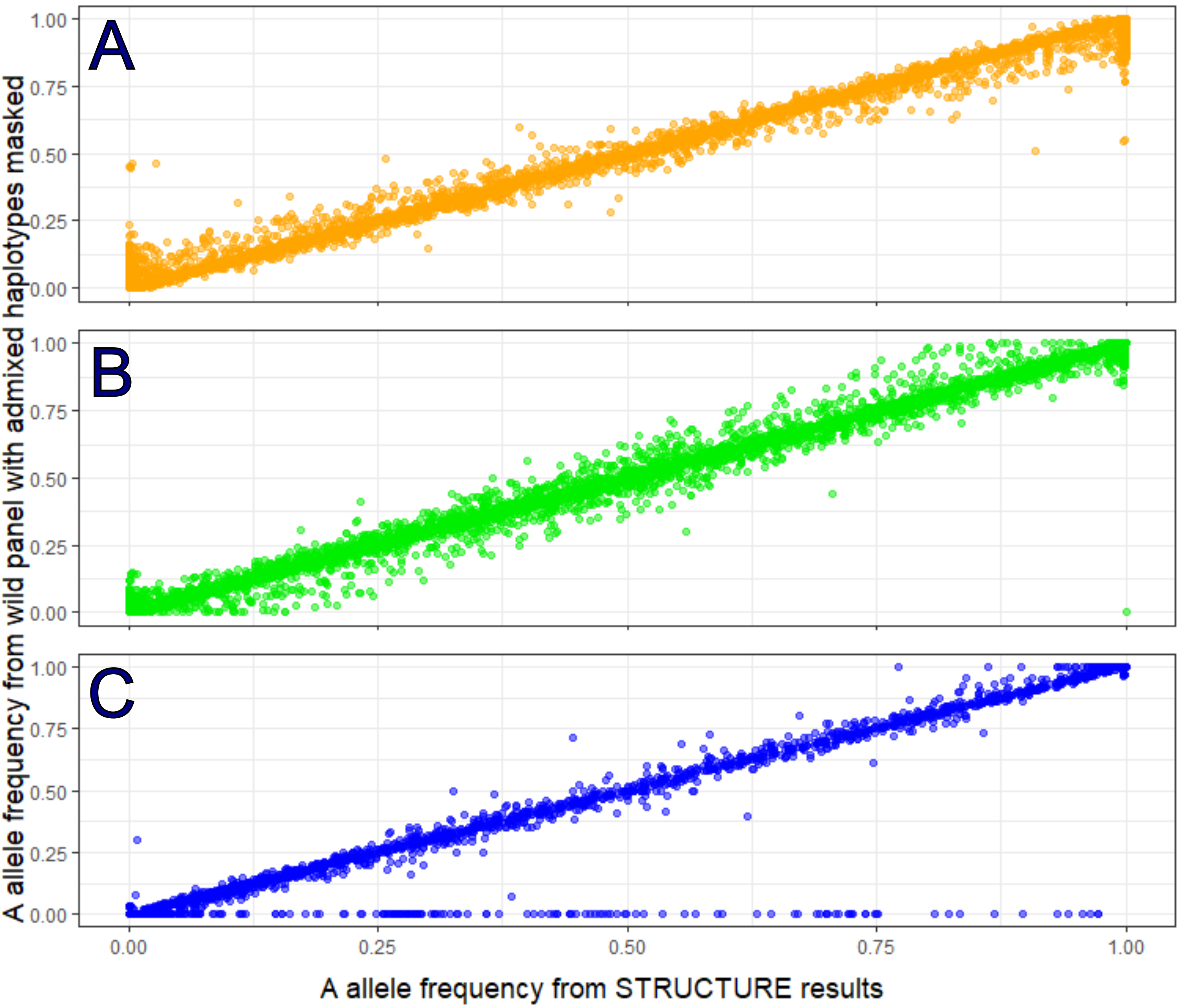

Figure S2: Frequency of the A allele assignments from results of STRUCTURE vs. A allele frequency assignments from the wild *Malus* panel for (A) *M. sieversii* and/or *M. orientalis*; (B) *M. sylvestris*; (C) tiny-fruited *Malus* species collectively named "exotic *Malus*".
