# Supplementary material for: Identifying the contributions of progenitor Malus species to cultivated apple (M. domestica) using 20K SNP array data
Source: BMC Genomics. 2026 Jun 11;27:536. doi: 10.1186/s12864-026-13023-z (PMC13255378; doi:10.1186/s12864-026-13023-z)
Supplement: Supplementary file 3 — Supplementary Material 3. [file 12864_2026_13023_MOESM3_ESM.pdf]

Identifying the contributions of progenitor *Malus* species to cultivated apple (*M. domestica*) using 20K SNP array genotypic data

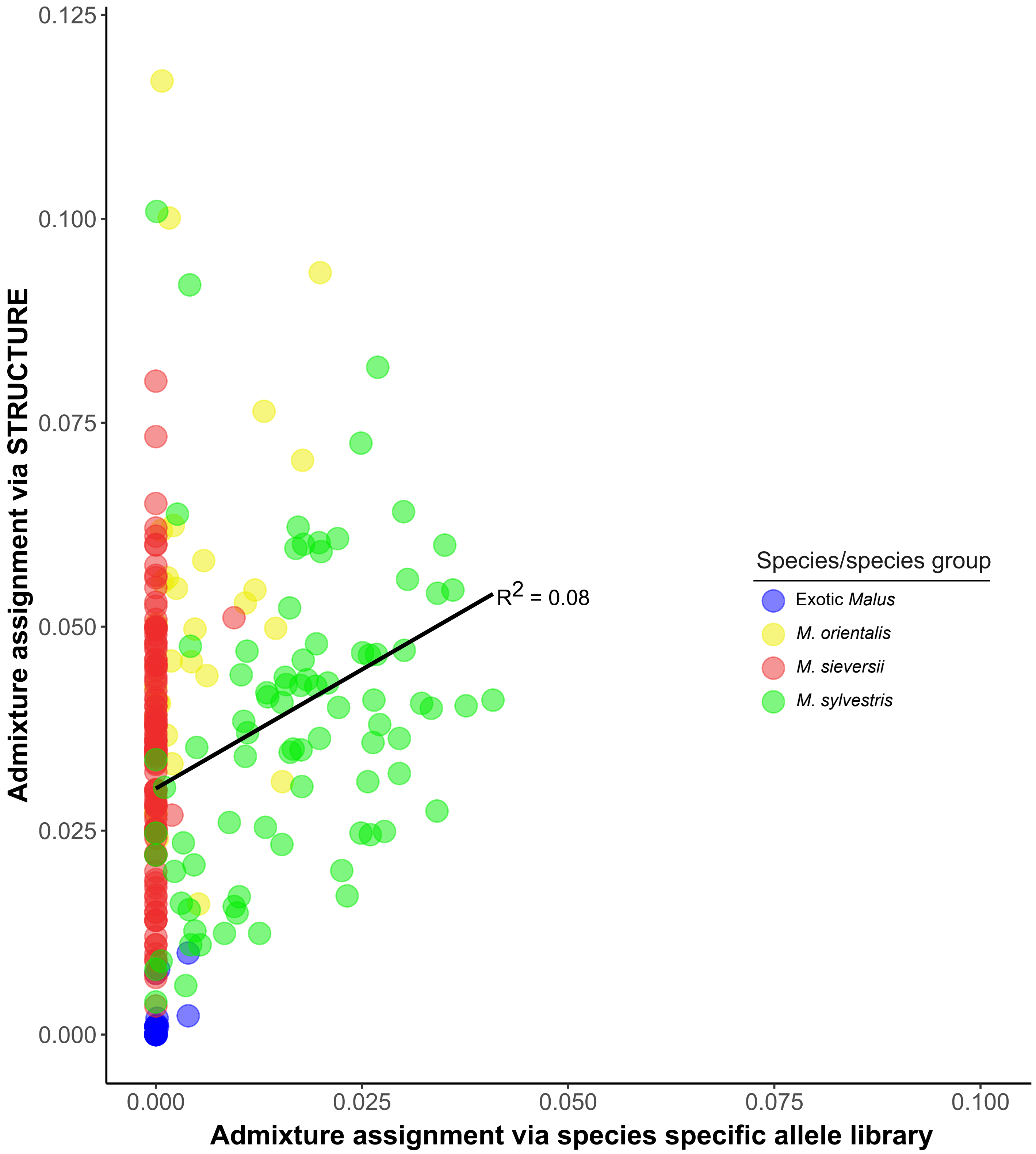

Figure S3: Proportion of the genotypic profiles of wild accessions included in the wild panel attributed to species other than their previously recorded or indicated species (i.e., the admixed proportion) estimated by ancestry attribution via the species-specific SNP allele library (x-axis) compared to that estimated by STRUCTURE analysis (y-axis).
