# Supplementary material for: Identifying the contributions of progenitor Malus species to cultivated apple (M. domestica) using 20K SNP array data
Source: BMC Genomics. 2026 Jun 11;27:536. doi: 10.1186/s12864-026-13023-z (PMC13255378; doi:10.1186/s12864-026-13023-z)
Supplement: Supplementary file 4 — Supplementary Material 4. [file 12864_2026_13023_MOESM4_ESM.pdf]

Identifying the contributions of progenitor *Malus* species to cultivated apple (*M. domestica*) using 20K SNP array genotypic data

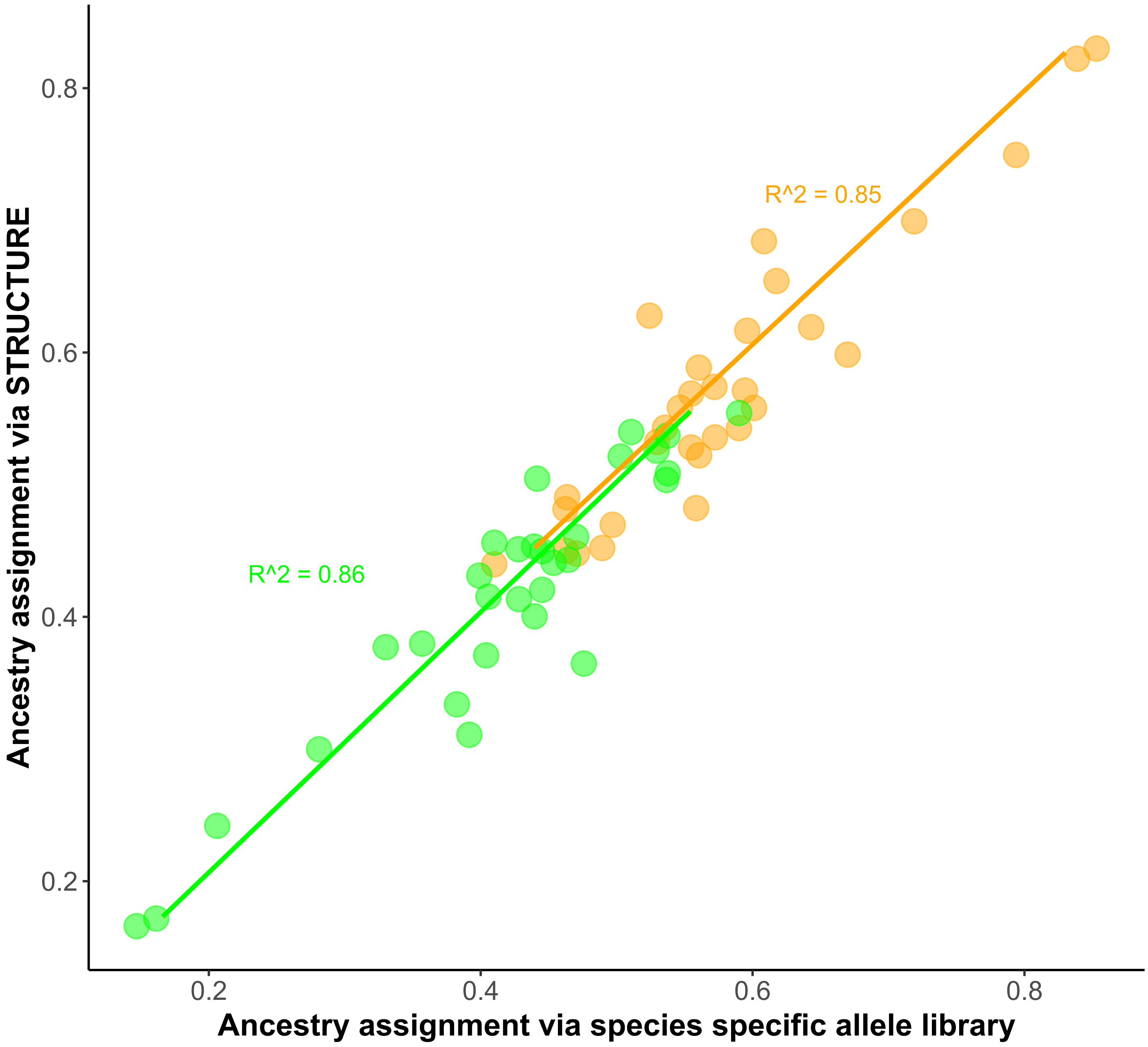


**Figure S4:** Comparison of the proportions of genotypic profiles of *M. domestica* cultivars attributed to *M. sylvestris* (green) and *M. sieversii* and/or *M. orientalis* (orange) between results from application of the species-specific allele library (x-axis) and from STRUCTURE (y-axis).
